# Potential Trace Metal–Organic Complexation in the Atmosphere

**DOI:** 10.1100/tsw.2002.132

**Published:** 2002-03-21

**Authors:** Hiroshi Okochi, Peter Brimblecombe

**Affiliations:** School of Environmental Sciences, University of East Anglia, Norwich NR4 7TJ, UK

**Keywords:** metal, organic compounds, complexation, carboxylic acid, dicarbonyl, humic-like substances

## Abstract

It is possible that metal–organic complexation enhances the uptake of gaseous organic compounds and the solubility of metals in aerosols and atmospheric water. We investigated potential atmospheric organic ligands and the enhanced uptake of hydroxy-, oxo-, and dicarboxylic acids as well as dicarbonyls into atmospheric aqueous aerosol. We examined complexation with transition metals (iron, manganese, nickel, copper, zinc) and lead on the basis of available references and our experimental data. Humic-like substances are most likely ligands in the atmosphere, although this is a poorly characterized material. A number of polycarboxylic acids and hydroxy forms (e.g., citric and tartronic acids) effectively complex metals such as copper in atmospheric aerosols. The simple equilibrium model calculations show that the effect of the complexation on the gas–aqueous phase partition of gaseous atmospheric ligands is quite small for the ligands with the high physical Henry’s law constants, e.g., dicarboxylic acids represented by oxalic acid, even if they have high affinity with metal ions. The lower Henry’s law constants of the α-dicarbonyls, such as glyoxal and methylglyoxal, mean that the complexation could lead to profound increases in their partition into the aqueous phase. Despite quantum mechanical arguments for copper–glyoxal complexes, experiments showed no evidence of complexation between either hydrated or unhydrated α-dicarbonyls and the cupric ion. By contrast the β-dicarbonyl, malondialdehyde, has properties that would allow it to partition into atmospheric water via the complexation with metal ions under some conditions.

## INTRODUCTION

Metals can be both essential nutrients and toxins, so their mobilization in the natural environment has been of interest for a long time. The speciation of metals is important and has a profound effect on their biological availability, as it influences solubility, not only in the aqueous phase but also in cellular matrices and tissue. In marine and aquatic chemistry, metals are not entirely free and may occur as a range of complexes[1,2]. A limited number of studies of the speciation of metals in aqueous atmospheric systems suggest that complex formation is also significant in the atmosphere[3].

Aqueous transition metal ions, in particular iron and manganese, have long been known to play a role in aqueous sulfite oxidation through their action as aqueous-phase catalysts. The complexation of metal ions could also affect the ability of metals in aerosols to act as catalysts[3]. Ligands can stabilize metal ions in different oxidation states, and the complexation leads to other reaction pathways, i.e., photochemical reaction. The formation of $H_2O_2$ and OH radicals, which are the most reactive oxidizing species for the transformation of various atmospheric inorganic and organic pollutants, by the photochemical cycling of the metal–organic acid complexes, in particular iron(III)–oxalate complex, in sunlight is well recognized (e.g., [4,5]). The photochemical reaction of the complexes of dicarboxylic and $\alpha$-keto acids such as oxalic, pyruvic, and glyoxalic acids with metal ions in atmospheric water leads to the depletion of these organic acids in the atmosphere.

The potential to bind substantial amounts of the metals as complexes can influence the solubility of the metals and of organic compounds in the aqueous phase or alter the availability of the metals for biological or catalytic interactions. It is also possible, once metals become bound to ligands, that they could become associated with organic phases in the atmosphere. There are no observations on this in atmospheric aerosols, but in model aqueous systems it is seen in the way in which metal complexes move into octanol; e.g., benzyl- and n-hexadecyl-malonato copper(II) show very different toxicity and partition into octanol[6]. The reactions of metals and ligands in solution could also enhance the partition of organic gases into the aqueous phase.

This paper aims to explore these possibilities on the potential metal ligand complex formation offered by likely atmospheric ligands by reviewing references and some experimental estimations for the stability constants of atmospherically important $\alpha$-dicarbonyls.

## THE FORMATION OF METAL-ORGANIC COMPLEXES IN ATMOSPHERIC WATER

There are many ways in which the degree of metal complex formation can be significant within the atmospheric aqueous phase, such as aerosols, cloud, fog, rain, and dewwater. In essence this requires that a substantial amount of either the metal or the ligand becomes complexed. Additionally, complex formation represents a pathway for enhancing the partition of organic materials into the aqueous atmospheric aerosol or the dissolution of the metals.

Let us consider briefly the dissolution of a monobasic organic acid gas, HL, into the atmospheric aqueous phase. Under ideal conditions, its activity and concentration are almost equal (i.e., low ionic strength); the process can be described in terms of Henry's law. For simplicity, charges are omitted.

$$HL_{(g)} \leftrightarrow HL_{(aq)} \qquad K_H = [HL_{(aq)}]/p_{HL} \qquad (1)$$

$$HL_{(aq)} \leftrightarrow H + L \qquad K_{a1} = [H][L]/[HL_{(aq)}] \qquad (2)$$

where $[HL_{(aq)}]$ and [L] are aqueous concentrations of the undissociated and dissociated ligand (mole per liter of liquid), [H] the concentration of hydrogen ion (mole per liter of liquid), and $K_H$ the Henry's law constant (mole per liter of liquid per atmosphere, M atm$^{-1}$), respectively. The symbol $p_{HL}$ is the partial pressure of HL (in atm) and equal to $[HL_{(g)}]RT$, where $[HL_{(g)}]$ is the gas-phase concentration (mole in liter of air), R the gas constant, and T the temperature Kelvin. The ligand can complex with a metal ion, M, in solution:

$$M + L \leftrightarrow ML \qquad K_{ML} = [ML]/[M][L] \qquad (3)$$

where $K_{ML}$ is the stability constant for the reaction and [ML] and [M] is the complexed and uncomplexed metal concentration (mole per liter of liquid), respectively.

In situations where the metal concentration is substantially lower than the ligand concentration, we can determine the amount of the organic gas required in the air to cause half the metal ion in the aqueous phase to be complexed by combining with the Henry's law constant and liquid water content. This is a useful guide for representing ability of organic compounds to complex metals in atmospheric water, as shown in Table 1. Half of the metal will be complexed when $[L] = 1/K_{ML}$ and [ML] is much smaller than [L], so the total concentration of the ligand in the atmosphere, $[HL]_T$, can be given by

$$\begin{aligned}[HL]_T &= [HL_{(g)}] + [HL_{(aq)}]_T \bullet LWC \\ &= [HL_{(g)}] + ([HL_{(aq)}] + [L] + [ML]) \bullet LWC \\ &\approx [HL_{(g)}] + ([HL_{(aq)}] + [L]) \bullet LWC \\ &= 1/K_{ML}\ \{[H]/(K_{a1}K_HRT) + ([H]/K_{a1} + 1) \bullet LWC\} \qquad (4)\end{aligned}$$

where LWC is liquid water content (liter of liquid in liter of air). There is also the potential for metal ions to become bound to $OH^-$, but this is fairly small under typical slightly acidic atmospheric conditions. Some potential ligands may be dibasic (e.g., oxalic) or even tribasic (e.g., citric acid). Under these situations it is more complicated to determine the amount of ligand required to complex the metal, although the equations can be readily written. Further assuming that the fractions except 1:1 type complex, i.e., $ML_2$, $ML_3$, MHL, $M(HL)_2$, and so on are negligible, Eq. 4 can be rewritten for dibasic acids:

$$[H_2L]_T \approx 1/K_{ML}\ \{[H]^2/(K_{a1}K_{a2}K_HRT) + ([H]^2/(K_{a1}K_{a2}) + [H]/K_{a2} + 1) \bullet LWC\} \qquad (5)$$

A different situation can arise when the metal concentration is close to that of the ligand concentration in solution. In such a system the amount of aqueous ligand will be the sum of bound and unbound ligand, i.e., $[HL_{(aq)}] + [L] + [ML]$ for a monobasic acid. This shifts the balance of the organic speciation in solution as alluded to in the introduction and could enhance partition into the liquid phase. We can write a pseudo Henry's law constant ($K_H^*$) to describe the overall dissolution:

$$\begin{aligned}[HL]_T &= [HL_{(g)}]\ (1 + K_H^*\ RT \bullet LWC) \\ K_H^* &= K_H\ \{1 + K_{a1}/[H](1 + K_{ML}\ [M])\} \qquad (6)\end{aligned}$$

It is clear that significant enhancement of gaseous ligand solubility through the complexation requires that $K_{a1}/[H](1 + K_{ML}\ [M]) > 1$. However it also requires that $K_H$ is not so large that the ligand already partitions into the aqueous phase in the atmosphere. This would mean that the enhancement is only relevant for $K_H$ perhaps $<10^6$ M $atm^{-1}$, although this is dependent on the amount of liquid water in the atmosphere. This process is again complicated where there are more complexes than the simple 1:1 type treated above. However, low ligand concentrations make these relatively unimportant. For simplicity, complexes except those 1:1 type are omitted. In the case of dibasic acids, it is worth considering two kinds of 1:1 type complex, i.e., MHL and ML, such that a pseudo Henry's law constant for dibasic acids can be given as:

$$\begin{aligned}[H_2L]_T &= [H_2L_{(g)}]\ (1 + K_H^{\ddagger}\ RT \bullet LWC) \\ K_H^{\ddagger} &= K_H\ \{1 + K_{a1}/[H](1 + K_{MHL}\ [M]) + K_{a1}K_{a2}/[H]^2(1 + K_{ML}\ [M])\} \qquad (7)\end{aligned}$$

where $K_{MHL}$ and $K_{ML}$ are stability constants of HL and L for metal ion, M, respectively. The enhanced dissolution of dibasic acids into the aqueous phase via the complexation also requires that $K_{a1}/[H]\ (1 + K_{MHL}\ [M]) > 1$ and/or $K_{a1}K_{a2}/[H]^2\ (1 + K_{ML}\ [M]) > 1$.

**TABLE 1**
**Stability Constants of Low Molecular Organic Compounds as Candidate Ligands in the Air**

| Candidates | Acid Dissociation*1 | | | Log $K_{ML}$ for Transition Metals*1 | | | | | | | $K_H$*2 | CRC50 (ppb)*3 | |
|---|---|---|---|---|---|---|---|---|---|---|---|---|---|
| | $pK_{a1}$ | $pK_{a2}$ | $pK_{a3}$ | $Mn^{2+}$ | $Fe^{2+}$ | $Fe^{3+}$ | $Ni^{2+}$ | $Cu^{2+}$ | $Zn^{2+}$ | $Pb^{2+}$ | (M $atm^{-1}$) | Cu(II) | Fe(III) |
| **Carbonyls** | | | | | | | | | | | | | |
| C2 Glycolaldehyde | | | | — | — | — | — | — | — | — | $4.14 \times 10^4$ | | |
| C2 Glyoxal | | | | — | — | — | — | — | — | — | $3.6 \times 10^5$ | | |
| C3 Methylglyoxal | | | | — | — | — | — | — | — | — | $3.71 \times 10^3$ | | |
| C4 Biacetyl | | | | — | — | — | — | — | — | — | $7.4 \times 10^1$ | | |
| C3 Malondialdehyde | 4.7 | | | — | — | — | 2.5 | 4.0 | — | — | $1.6 \times 10^{4\ BC}$ | 3.5 | |
| C5 Acetylacetone | 9.0 | | | 4.2 | 5.1$^b$ | 9.8$^b$ | 6.0 | 8.3 | 5.1 | 7.3$^b$ | $4.3 \times 10^{2\ WV}$ | 117 | 3.7 |
| **Oxocarboxylic acids** | | | | | | | | | | | | | |
| C2 Glyoxylic acid | 3.5 | | | — | — | — | 1.6 | — | 1.6 | — | $9 \times 10^{3\ SH}$ | | |
| C3 Pyruvic acid | 2.6 | | | 1.9 | — | — | 1.7 | 2.2 | 1.9 | 2.3 | $3.11 \times 10^5$ | 16 | |
| C4 Acetoacetic acid | — | | | — | — | — | — | — | — | — | — | | |
| **Hydroxylated acids** | | | | | | | | | | | | | |
| C2 Glycolic acid | 3.8 | | | 1.6 | 1.9 | 3.5 | 2.3 | 2.9 | 2.4 | 2.6 | $5.0 \times 10^{5\ WV}$ | 3.4 | 27 |
| C3 Lactic acid | 3.9 | | | 1.4 | — | — | 2.2 | 3.0 | 2.2 | 2.8 | $1.0 \times 10^{5\ WV}$ | 3.4 | |
| C3 Tartronic acid | 2.2$^a$ | 4.7$^a$ | | — | — | — | 4.3$^a$ | 6.2$^a$ | 4.1$^a$ | — | $1 \times 10^{18\ SH}$ | $2.3 \times 10^{-3}$ | |
| C4 Malic acid | 3.5 | 5.1 | | 3.3 | 3.5$^a$ | 8.4$^a$ | 4.1$^a$ | 4.3$^a$ | 3.3 | — | $1.7 \times 10^{11\ WV}$ | 0.28 | $2.2 \times 10^{-5}$ |
| C4 Meso-Tartaric acid | 3.2 | 4.9 | | — | — | 8.0 | — | 4.4 | — | 4.5 | $1.0 \times 10^{18\ SH}$ | 0.18 | $4.4 \times 10^{-5}$ |
| C4 D-Tartaric acid | 3.0 | 4.4 | | 3.4 | 2.6 | 6.9 | 3.3 | 4.1 | 3.8 | 3.8 | $1.0 \times 10^{18\ SH}$ | 0.24 | $3.9 \times 10^{-4}$ |
| C6 Citric acid | 3.1 | 4.8 | 6.4 | 5.2 | 5.7$^a$ | 13.5$^a$ | 6.7 | 7.2$^a$ | 6.2 | 5.4 | $6.3 \times 10^{11\ WV}$ | $6.5 \times 10^{-3}$ | $3.3 \times 10^{-9}$ |
| **Dicarboxylic acids** | | | | | | | | | | | | | |
| **<Normal saturated diacids>** | | | | | | | | | | | | | |
| C2 Oxalic acid | 1.3 | 4.3 | | 4.0 | 4.2 | 9.4 | 5.2 | 6.2 | 4.9 | 5.9 | $7.24 \times 10^8$ | $1.9 \times 10^{-3}$ | $1.2 \times 10^{-6}$ |
| C3 Malonic acid | 2.8 | 5.7 | | 3.3 | 3.3 | 9.4 | 4.1 | 5.7 | 3.8 | 3.7 | $3.7 \times 10^{6\ WV}$ | $3.0 \times 10^{-2}$ | $5.9 \times 10^{-6}$ |
| C4 Succinic acid | 4.2 | 5.6 | | 2.3 | 2.4$^c$ | 8.8 | 2.3 | 3.3 | 2.5 | 3.2 | $2.7 \times 10^{9\ WV}$ | 7.4 | $2.4 \times 10^{-5}$ |
| C5 Glutaric acid | 4.3 | 5.4 | | 2.1 | — | 8.7 | 2.0 | 3.2 | 2.5 | 3.2 | $3.20 \times 10^{9\ WV}$ | 6.6 | $3.1 \times 10^{-5}$ |
| C6 Adipic acid | 4.4 | 5.4 | | — | — | — | 2.5 | 3.4 | 2.4 | 3.2 | $2.18 \times 10^{8\ WV}$ | 4.2 | |
| C7 Pimelic acid | 4.5 | 5.4 | | 2.1 | — | — | 2.1 | 3.1 | 2.2 | 3.5 | $1.14 \times 10^{7\ WV}$ | 8.9 | |
| **<Branched saturated diacids>** | | | | | | | | | | | | | |
| C4 Methylmalonic acid | 3.0 | 5.8 | | — | — | 9.4 | 3.5 | 5.8 | 3.4 | — | — | | |
| C5 Ethylmalonic acid | 3.1 | 5.9 | | — | — | — | 3.5 | 5.8 | 3.4 | — | — | | |
| C5 Dimethylmalonic acid | 3.2 | 6.1 | | — | — | — | 2.8 | 5.5 | 3.1 | — | $3.7 \times 10^{5\ WV}$ | 0.10 | |
| C5 Methylsuccinic acid | 4.1 | 5.8 | | — | — | — | — | — | — | — | — | | |
| C6 Isopropylmalonic acid | 2.9 | 5.9 | | — | — | — | 3.5 | 6.3 | 3.8 | — | — | | |
| C6 2-Methylglutaric acid | 4.5 | (5.7) | | — | — | — | — | — | — | — | — | | |
| **<Unsaturated diacids>** | | | | | | | | | | | | | |
| C4 Maleic acid | 1.9 | 6.3 | | 2.7 | — | — | 2.9 | 4.3 | 2.9 | 4.1 | $8.0 \times 10^{7\ WV}$ | 2.8 | |
| C4 Fumaric acid | 3.1 | 4.5 | | 2.0 | — | — | — | 2.5 | — | — | $3.0 \times 10^{5\ WV}$ | 10 | |
| C5 Methylmaleic acid | 2.4 | 6.0 | | 2.8 | — | — | 2.7 | 4.3 | 2.7 | 4.2 | — | | |
| **<Aromatic/cyclic diacids>** | | | | | | | | | | | | | |
| C8 Phthalic acid | 3.0 | 5.4 | | 2.7 | — | — | 3.0 | 4.0 | 2.9 | — | $5.0 \times 10^{7\ WV}$ | 0.88 | |
| C8 cis-Norpinic acid | 4.3 | 5.5 | | — | — | — | — | — | — | — | — | | |
| C9 Pinic acid | 4.5 | 5.5 | | — | — | — | — | — | — | — | — | | |

*1 The values at 25°C unless otherwise specified from Martell and Smith[10], IUPAC SC-database[11], and Howell and Fisher[12].

*2 The values at 25°C unless otherwise specified from ChemFinder[82], Interactive PhysProp Database Demo[83], and Sander[84].

*3 The ligand's atmospheric Concentration (ppb) Required to lead to the Complexation of 50% of total copper (II) or total iron (III).

$^a$ = 20°C; $^b$ = 30°C; $^c$ = 37°C.

When necessary, constants at ionic strength (I) zero were calculated using the Davies equation for < I = 1.0 M, and a table, which was shown in Morel and Hering[13], for > I = 1.0 M. Estimated Henry's law constants as follows: WV = the ratio of water solubility to vapor pressure; SH = estimated by Saxena and Hildmann[7]; BC = estimated using the bond contribution method proposed by Meylan and Howard[85].

## CANDIDATE ORGANIC LIGANDS IN THE ATMOSPHERE

Likely organic ligands could be present as gases or aerosols in the atmosphere. Water-soluble organic compounds (WSOC) account for 20 to 70% of total carbon in the atmospheric aerosol[7]. A range of possible organic compounds has been suggested as contributing to the water soluble material in atmospheric particles[7,8]. More recently polyols/polyethers, aliphatic mono-/dicarboxylic acids (many hydroxylated), and highly unsaturated polyacidic compounds (probably fulvic/humic acid) have been found to account for 77% of WSOC in fog water samples, using a combination of chromatographic separation, $^{1}$H NMR, and TOC analysis[9]. Aldehydes, esters, organic nitrates, aliphatic nitro-compounds, amines, and amino acids did not appear as major contributors to WSOC.

Table 1 lists some stability constants of candidate organic compounds in the atmosphere. The compounds were chosen generally based on suggestions by Saxena and Hildemann[7] and Decesari et al.[9]. Some compounds listed in Table 1 have not been identified in the atmosphere yet, although future improvements in analytical methods will increase the likelihood. Many of the stability constants are from Martell and Smith[10] and IUPAC SC-database[11]. When necessary, the constants at zero ionic strength were calculated using the Davies equation for ionic strength <1.0 M and corrected for ionic strength >1.0 M according to Morel and Hering[13]. Approximations were also required for the Henry's law constants, as many of these compounds have exceedingly large Henry's law constants or are involatile, such that experimental determination is difficult. Unfortunately, theoretical estimations from methods such as bond contribution are often unreliable for polar compounds, but our calculations are insensitive to the Henry's law constants once they are greater than about $10^{6}$ M atm$^{-1}$.

Two columns at the right in Table 1 give the concentrations required to lead to 50% complexation of trace amounts of copper(II) and iron(III) by a given ligand (this parameter is abbreviated to CRC50). The CRC50s were calculated from Eq. 4 or 5 under the condition of pH 5.0 and LWC $10^{-7}$, which is typical of a fog droplet[14]. Copper was chosen because of its strong affinity for naturally occurring organic matter and ligands in general. It is often found as organically complexed forms in atmospheric aerosols and rain water[3,15,16]. Iron was chosen because it is a common element, and in the ferric state the high charge can lead to high stability constants.

Fig. 1 shows the relationship between $pK_a$ and the logarithm of stability constants of organic ligands, which are listed in Table 1 except for amino acids, with cupric ion. We can see binding of organic ligands with cupric ion increase with hydrogen binding. In general one can argue that simple monocarboxylic acids (e.g., formic and acetic) do not complex substantial amounts of metal under typical atmospheric conditions. The oxy and hydroxy acids are a little more effective but still require high gas-phase concentrations for this to occur. Oxalic, tartronic, and citric acids are more powerful ligands and can effectively complex copper(II) and iron(III) at low gas-phase concentrations. Amino acids and acetylacetone are not potential ligands in the atmosphere because they have higher $pK_a$s than the atmospheric pH ranges, but malondialdehyde (MDA) is acidic enough to form complexation with metal in these pH ranges.

We will treat various important classes of candidate compounds in more detail below.

## Oxo Compounds

### *Monofunctional Compounds*

Monocarboxylic acids and other monofunctional compounds (alcohols, aldehydes, etc.) complex only weakly with transition metal ions. Typically the $K_{a1}$ values are under $10^{-3}$ mol l$^{-1}$ for monocarboxylic acids, i.e., formic and acetic acids. They also have relatively low Henry's law

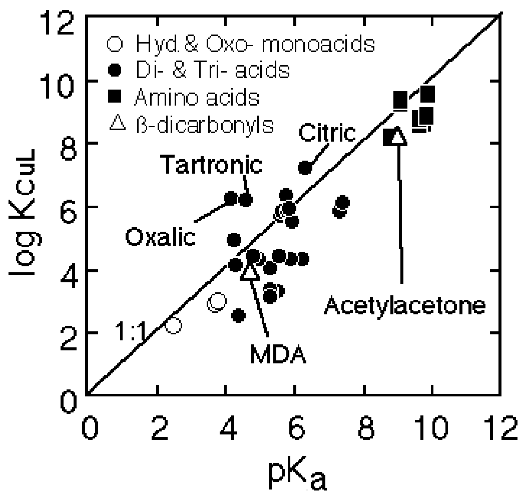

**FIGURE 1**. The relationship between $pK_a$ and the logarithm of stability constants of some organic ligands for the cupric ion as listed in Table 1 (except for amino acids, which are not listed).

constants (about $10^3$ M $atm^{-1}$ for the acids), so very high atmospheric concentrations are required if there is to be substantial complex formation with copper or iron. In the case of acetic acid, for example, this would be about 400 and 7 ppb for copper(II) and iron(III), respectively, which would seem possible in only the most extreme circumstances. Monocarbonyls tend not to bind strongly; for example, acetone has a very small stability constant of $10^{0.57}$ with cupric ion at 27°C and unknown ionic strength[11]. So unrealistically high concentrations of metal ion would also be required to enhance the partition of acetone into the aqueous phase.

## Dicarbonyls

The α-dicarbonyls, glyoxal, methylglyoxal, and biacetyl, are formed from the gas-phase oxidation of aromatic hydrocarbons such as toluene, m-xylene, p-xylene, 1,2,4- and 1,3,5-trimethylbenzene[17,18], and from the smouldering combustion of wood[19]. Extensive field measurements have made it clear that the high solubility and hydration of the α-dicarbonyls makes them important components of atmospheric water droplets such as cloud, fog, rain, and dew (e.g., [20,21]). These carbonyls play important roles in enhancing the uptake of $SO_2$ from ambient air into droplets through stabilization as α-hydroxyalkylsulfonates and ultimately acidification[22].

If α-dicarbonyls were to complex effectively with metal ions in the aqueous phase, it would enhance the dissolution of gaseous α-dicarbonyls but compete with sulfur(IV) in the formation of α-hydroxyalkylsulfonates. Weschler et al.[23] have suggested that the coordination of aldehydes to transition metal ions may also facilitate the oxidation of aldehydes because ligand bonding reduces the electron density on carbon, weakens the carbon–hydrogen bond, and promotes hydrogen abstraction. Oxidation of glyoxal, which leads to glyoxylic acid and OH radical followed by the formation of oxalic acid[24], would be of further interest because oxalic acid is also a powerful ligand for many transition metal ions in aqueous aerosols. There are theoretical indications (e.g., [25]) that the copper complex with glyoxal would allow for an easy transfer between the copper(I) and copper(II) states. *Ab initio* MO SCF calculations suggest that α-dicarbonyls might form complexes with alkali, alkali earth, and transition metal ions[26]. However, UV, $^1$H NMR, and metal NMR spectroscopy revealed little experimental evidence[27,28,29] for complexes with biacetyl (2,3-butadione). There are no measured stability constants of α-dicarbonyls with metal ions, so we tried to determine the stability constants of glyoxal and methylglyoxal with cupric ion using cupric ion selective electrode (see Appendix). We found no evidence of complexation between either unhydrated or hydrated forms of glyoxal and cupric ion. The result indicates no substantial proportion of glyoxal and methylglyoxal would be bound to copper(II) in the aqueous aerosol. It also seems unlikely that other α-dicarbonyls, e.g., biacetyl, would form complexes.

By contrast β-dicarbonyls, such as malondialdehyde (propanedial, $CHOCH_2CHO$) and particularly acetylacetone (pentan-2,4-dione, $CH_3COCH_2COCH_3$), are powerful ligands as shown in Table 1 because they form enolate anions in water. Neither of these β-dicarbonyls has been detected in the atmosphere, although malondialdehyde (MDA) is formed in smog chambers, via OH- and $O_3$-initiated photo-oxidation of 1,3-butadiene and 4-oxo-2-pentenal, the products of aromatic photochemical oxidation[30,31]. A further route for MDA production is the photo-oxidation of glycolaldehyde (hydroxyethanal) in solution[32]. An isomer of acetylacetone, levulinaldehyde (4-oxopentanal, $CH_3COCH_2CH_2CHO$), is known as an ozonolysis product of leaf waxes[33]. As shown in Table 1, the enol form of MDA is acidic enough to form at atmospheric pH values (i.e., $pK_a$ 4.46) and it forms relatively strong complexes with metal ions. The palladium(II) and chromium(III) chelates are stable enough to sublime[34], while the stability constants for copper(II) and nickel(II) are known[35]. MDA is also likely to have a relatively small Henry's law constant and enolization likely to restrict the hydrolysis that enhances the partition of many other aldehydes. This makes it possible for the enhanced uptake of gaseous MDA into aqueous phase via complexation where metal ions are abundant. We will discuss the possibility of the enhanced uptake of MDA into fog water via the complexation later. On the other hand, the enol form of acetylacetone will only dissociate under alkaline conditions, so its complexes are not likely to be important in the atmosphere (see Table 1).

## Dicarboxylic Acids

The dicarboxylic acids are strong acids and have high Henry's law constants and large stability constants. This has made them prime candidates for atmospheric ligands. Table 1 shows that very low concentrations, of just a few parts per trillion, of oxalic and malonic acids will lead to substantial trace metal complexation. The longer-chain acids are both weaker acids and do not bind as strongly to metal ions. Substituted malonic acids may complex even more strongly than the parent acid.

If metal ion concentrations approach typical concentrations of oxalic and malonic acids in solution, these acids will be largely present as oxalato and malonato metal complexes. Evidence from laboratory chemistry shows dicarboxylic acid complexes to be photoreduced. The photoreduction of copper(II) to copper(I) is most effective when it is present as oxalato complexes and much less so with succinato and malonato complexes[36]. The photoreduction of iron(III) oxalato and malonato complexes seems to have higher quantum yields than for complexes with succinic acid[37]. This may well explain the observations that oxalic and malonic acid appear to be depleted in rain[21], such that succinic acid becomes the dominant dicarboxylic acid in the Arctic[38]. This supports suggestions that the depletion of oxalic and malonic acids arises through increased photodegradation[21].

Some aromatic compounds are potential chelates, most notably those with ortho positioned oxo groups such as phthalic acid, which is known in remote aerosols[39] and can derive from naphthalene oxidation[40]. The calculations in Table 1 suggest that less than ppb levels of phthalic acid would complex substantial amounts of trace copper. Such complexation may well assist the photodegradation of the aromatic ring[5]. In forested areas, pinic acids (i.e., dicarboxylic acids based on cyclobutane: e.g., pinic acid [or 3-carboxy-2,2-dimethylcyclo-butylethanoic acid] and norpinic acid [or 2,2-dimethyl-trans-1,3-cyclobutanedicarboxylic acid]) are potential ozonolysis products of monoterpenes. Although there are no direct measurements of the stability constants of these acids with transition metals, comparison with similar chain-length dicarboxylic acids would suggest that they are weakly bound to metals. Nevertheless forest fire aerosols could well be alkaline from calcium or potassium salts[41] and combine with high concentrations of forest-derived acids to lead to some complex formation.

## *Oxo and Hydroxy Acids*

While acids are the most obvious complexing agents, some other highly oxygenated compounds show an ability to form complexes. For example, while alcohols are usually poor complexing agents, glycerol (propanetriol) shows a tendency to complex with softer metals such as lead: K(PbGlyc) = $10^{1.5}$. This is too weak, though, to be generally important in the atmosphere.

Hydroxy and oxo monobasic acids show greater potential to form complexes. Lactic (2-hydroxy propanoic), β-hydroxy propanoic (3-hydroxy propanoic), and pyruvic (2-oxopropanoic) acids are relatively volatile and exhibit high Henry's law constants, but the available stability constants are smaller than with the C-2 and C-3 dicarboxylic acids. In some circumstances, where their concentrations were in the ppb range, complexes would be formed, and the possibility of photodegradation via iron(III) complexes has been recognized[5]. The β-positioned oxo group acid in acetoacetic (3-oxo butanoic) acid might well allow relatively high stability constants, but there are no available data for such complexes.

The hydroxy polybasic acids are less volatile. Tartronic (hydroxy propanedioic), tartaric (2,3-dihydroxy butanedioic), and citric (2-hydroxy-1,2,3-propanetricarboxylic) acids are relatively strong acids and readily form complexes with transition metals. These compounds have not been reported in atmospheric aerosols, although their presence would not come as particularly surprising, as they are stable and the latter two are important products of biochemical processes. We can see from the CRC50 columns in Table 1 that they lead to iron(III) and copper(II) complexes at relatively low concentrations. Tartaric and citric acids become more easily photoreduced once bound to iron(III) through Fenton-type processes. Abrahamson et al.[37] found that tartaric acid had high quantum yields, even higher than citrate complexes. Acetone was identified as the ultimate product of such decarboxylations. The photo-oxidation of metals in simulated atmospheric water is also affected by organic materials such as citrates and tartrates. Zhang[42] described the photoinduced oxidation of chromium(III) in the presence of these organic materials and metals such as iron and manganese.

Aromatic hydroxy aldehydes and acids (e.g., salicylic acid and salicylaldehyde) are good complexing agents, but the dissociation constants of the acids are too small to allow complex formation in acid solution (e.g., $pK_a$ for salicylic acid is about 13).

## Nitrogen Compounds

Nitrogen compounds are also potential ligands. Ammonia complexes are well known, but in acidic solution this ligand will be protonated and therefore unlikely to form strong complexes. Even under favorable conditions, let us say at pH 6.5 in an aerosol with $10^{-4}$ mol $l^{-1}$ ammonia, we would expect <1% of trace levels of copper(II) to be complexed as $Cu(NH_3)^{2+}$ in solution (assuming the stability constant at $10^{4.13}$). Cyanides are found at relatively high concentration in forest fire plumes[43,44]; and though cyanide forms stable complexes, it is likely to remain protonated at atmospheric pH values. Alkylamine ligands and chelates such as cadaverine (1,5-diaminopentane)[45] may be found in the atmosphere from oceanic sources, but again complex formation would be limited by protonation.

There is a range of less volatile organic nitrogen compounds in the atmosphere, such as the amino acids. The total dissolved free amino acids (predominantly serine and glycine) are found in surprisingly high concentrations (micromolar) in marine rain samples and have been found at concentrations similar to inorganic nitrogen species[46,47]. Although amino acids can be strong organic ligands, they are only able to act in this way at pH >9.

Urea and uric acid are also found in aerosols[48,49] and have the ability to form complexes, although the measured stability constants with urea are low (e.g., with copper[II] only $10^{-0.7}$ at I = 0.20 M)[11]. The complexes of uric acid with chromium(III) and especially copper(II) inhibit its breakdown in allantoin and urea[50].

Nitrated phenols are formed by atmospheric photochemical reactions of aromatic compounds such as benzene, toluene, and cresols with OH radical and nitrogen compounds (e.g., [51]). Some nitrated phenols such as 2-nitrophenol, 4-nitrophenol, 3-methyl-2-nitrophenol, 3-methyl-4-nitrophenol, 4-methyl-2-nitrophenol, 2,4-dinitrophenol, DNOC (2-methyl-4,6-dinitrophenol), and dinoseb (2-sec-butyl-4,6-dinitrophenol) are detected at nanomolar levels in fog and rain water[52,53], but there are few available stability constants of these compounds with metal ions. The dissolution of 2,4-dinitrophenol into droplets might be enhanced via the complexation because its $K_H$ is $3.5 \times 10^3$[54], combined with a fair ability to form complexes with copper ($pK_a$ is 3.93 at 25˚C and I = 0.10 M)[11]. Mononitrophenols (e.g., 2- and 4-nitrophenol) are less likely to act as ligands under atmospheric conditions because their $pK_a$s are about 7[11].

## Macromolecular Ligands

Macromolecular ligands, such as humic or fulvic acids, are able to bind very strongly with metal ions and are abundant within environmental systems. They are often seen as explaining the speciation of metal ions in aquatic and marine environments, and about 90% of the freshwater organic component has been considered to be macromolecules or particles[1]. Fulvic acid is generally assigned the range of 500 to 2,000 Da and humic acid 2,000 to 5,000 Da. The lower molecular weight fraction of fulvic acid has a higher content of simple aromatic compounds with attached carboxy and/or hydroxy groups such as salicylic acid and phthalic acid, whereas the higher molecular weight fractions have a greater aromatic character and/or π-conjugation. The conditional stability constants of fulvic acid from a northern coniferous forest soil for aluminum(III) ranged from $10^{4.4}$ to $10^{5.3}$ and increased from the lower to the higher molecular weight fraction[55]. For the lower molecular fraction of fulvic acid (750 Da), the conditional stability constant for the fulvic acid–copper(II) complex has been reported to be $10^{5.9}$[56]. Complexation of the lower molecular weight fraction of fulvic acid with metal ions such as iron(III) and aluminum(III) promotes transformation to a higher molecular fraction (e.g., [57]).

Much of the work with these macromolecules relates to their behavior in marine and aquatic systems, where pH values are higher than found in the atmosphere. The binding strength of bivalent trace metals with humic substances follows the order: Cu > Ni > Pb > Cd > Zn >Mn[58]. For example, the stability constants of zinc(II) and cadmium(II) with humic acid at pH 5 are reported to be $10^{5.6}$ and $10^{5.5}$, respectively[59]. In natural waters, alkaline earth metal ions such as calcium compete with the trace heavy metal ions because they are generally present in large excess over the heavy metal ions and have relatively high affinities with humic material. Calcium/humate material stabilities range from $10^{2.6}$ to $10^{4.1}$, depending on the pH, the analytical method, the origin of the humic material, and the ionic strength[59]. In the presence of competing ions like calcium, the association of trace heavy metals with the humic material can be understood by incorporation of an additional equilibrium reaction for calcium with the humic/fulvic acids[59]. The broad range of measured complexation constants between fulvic acid and copper(II), $10^5$ to $10^{14}$ at pH 8, decrease to much lower values of $10^4$ to $10^{5.5}$ at pH 4[1]. Increasing protonation will be likely to reduce their ability to form complexes probably because of the changing of conformation from an extended shape at neutral and alkaline pH values to a coiled configuration under low pH conditions[60]. This may mean that metals will bind to humic and fulvic acids in a way that indicates their removal is hindered.

Humic-like compounds are also found in aerosol, rain, and fog water[61,62,63,64]. Likens and Galloway[62] have reported that dissolved macromolecular organic compounds (>1,000 Da) accounted for 35 to 43% of total organic carbon in precipitation. Of the organic carbon in aerosol collected in German urban and rural areas, 10% and more has been reported to be attributed to humic-like compounds[63]. Krivácsy et al.[65] indicate that these may play a role in cloud condensation like ammonium sulphate. The origins of humic-like compounds in fog water in Po Valley, Italy and in aerosol collected in Germany are both thought to be biomass combustion[66,63]. Despite the great difficulty in modeling humic-like substances in the air, experiments have shown that much copper is readily bound to organic complexants, terrestrial in origin, with potassium values in the order of $10^{11}$ to $10^{14}$. In rain water at pH 7.7, more than 99.9% of the copper(II) is organically complexed[3], although the relevance of these measurements to atmosphere pH values has still to be resolved. Cheng et al.[15] have investigated chemical speciation of some trace metals in rain water using a DPASV and ion-exchange method under atmospheric pH conditions. They have indicated that half of the zinc, lead, and cadmium in the soluble fraction was present as metal aqua ions and/or small inorganic complexes, whereas half of the copper in the soluble fraction was present as larger complexes of organic ligands. Copper in the soluble fraction was mainly present in the molecular weight range of 1,000 to 5,000 Da, which includes both fulvic and humic acids.

## CALCULATED PARTITION OF LIGANDS IN FOG WATER

Trial calculations using a simple equilibrium model illustrate the potential for enhanced uptake of gases into fog water. Let us consider the partition of oxalic acid because it is the most abundant dicarboxylic acid in the atmosphere and is a powerful ligand.

In a fog event with a given liquid water content (LWC), the concentration of total oxalic acid, $[H_2L]_T$, in the atmosphere can be given as follows as well as in Eq. 7:

$$[H_2L]_T = [H_2L]_{gas} + [H_2L]_{fog} \bullet LWC = [H_2L]_{gas} (1 + K_H^{\ddagger} RT \bullet LWC)$$
$$K_H^{\ddagger} = K_H \{1 + K_{a1}/[H](1 + \sum K_{MHL} [M]) + (K_{a1}K_{a2}/[H]^2)(1 + \sum K_{ML}[M])\} \quad (8)$$

where $K_{a1}$ and $K_{a2}$ are the first and second acid-dissociation constants and $K_{MHL}$ and $K_{ML}$ are stability constants of HL and L for each metal ion, M, respectively. The value $K_H^{\ddagger}$ is a total or operational Henry's law constant describing the overall dissolution. The summations (∑) are over the range of metal ions chosen (here iron, manganese, nickel, copper, zinc, lead). The mass balance of each metal ion in fog water can be given as follows:

$$[M]_{fog} = [M] + [MHL] + [ML] + [MOH] + [M(OH)_2]$$
$$= [M] \bullet D_M$$
$$D_M = \{1 + (K_{MHL}K_{a1}/[H] + K_{ML}K_{a1}K_{a2}/[H]^2) \bullet [H_2L]_{gas} \bullet K_H RT + \beta_{OH}K_w/[H] + \beta_{(OH)2}(K_w/[H])^2\} \quad (9)$$

where $\beta_{OH}$ and $\beta_{(OH)2}$ are stability constants of M(OH) and $M(OH)_2$, respectively, and $K_w$ is an ion product of water ($1.01 \times 10^{-14}$ at 25°C). For simplicity we ignore the formation of any hydroxide with metal ions except M(OH) and $M(OH)_2$. When the pH and the concentration of each metal ion in fog water are given, we can estimate the gas–aqueous partition of oxalic acid via the complexation as a function of the gas-phase concentration of oxalic acid before the formation of fog using the Eq. 8 and 9.

Fig. 2 shows the range of concentration of selected transition metals in rain and fog water measured at a wide variety of locations. We can see that some transition metals such as iron and

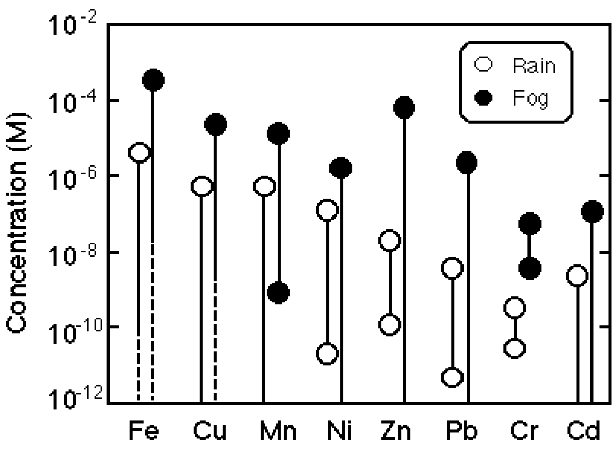

**FIGURE 2**. The concentration range of dissolved transition metal ions in rain and fog. Data source: for rain[67,68,69,70,71,72,73,74,75]; for fog[76,77,78,79,80,81]. We also used unpublished data for nickel, zinc, lead, and cadmium in fog water, which was collected in 1998 at the midslope of Mt. Oyama, Japan. The dotted line shows that the concentration is below detection limits, although they depend on the analytical methods.

zinc can be present in atmospheric water at submillimolar to millimolar levels at a maximum. These concentration levels of metals are almost equal to the concentration of atmospherically important organic ligands[7] listed in Table 1. Lower metal concentrations are seen in rain collected at oceanic and coastal sites, with higher levels occurring in fog water particularly in urban areas.

As an example, the enhanced uptake of oxalic acid into fog water via the complexation was estimated for the midslope of Mt. Oyama (1,252 m asl) in Japan, located 60 km southwest from Tokyo. We adopted iron, copper, manganese, nickel, zinc, and lead concentrations typical of the annual volume-weighted means in the soluble fraction in fog water collected in 1998. We here assumed that iron was present in the ferric state and other metals to be bivalent. Fig. 3 shows the partition of oxalic acid between gas phase and fog water and the fraction of metal–oxalate complex concentration to total oxalic acid concentration in fog water as a function of the gas-phase concentration of oxalic acid before the fog formation. The aqueous fraction of oxalic acid, $X_{aq}$, and the fraction of metal–oxalate complex, $\alpha_{com}$, were calculated following the Eq. 10 and 11, respectively:

$$X_{aq} = K_H^{\ddagger} RT \bullet LWC/(1 + K_H^{\ddagger} RT \bullet LWC) \times 100 \quad (10)$$

$$\alpha_{com} = (K_{a1}/[H] \bullet \sum K_{MHL}[M] + K_{a1}K_{a2}/[H]^2 \bullet \sum K_{ML}[M]) \div \{1 + K_{a1}/[H](1 + \sum K_{MHL}[M]) + (K_{a1}K_{a2}/[H]^2)(1 + \sum K_{ML}[M])\} \times 100 \quad (11)$$

As shown in Fig. 3a and 3c, oxalic acid almost completely partitions into fog water regardless of the pH, LWC, and the gas-phase concentration before the fog formation. In very low LWC and lower pH conditions, i.e., LWC $10^{-10}$ and pH 2 in the figure, oxalic acid is slightly present in the gas phase (Fig. 3a). The fraction of metal–oxalate complex, $\alpha_{com}$, increases as LWC increases and the gas-phase concentration and the pH decrease (Fig. 3b and 3d). However, the effect of the complexation on gas–aqueous phase partition of oxalic acid is quite small because the physical Henry's law constant of oxalic acid is high ($7.24 \times 10^8$ M atm$^{-1}$). Fig. 4 illustrates what happens to oxalic acid and to various trace metals in fog water with LWC $10^{-8}$ in the model calculation as shown in Fig. 3. At very low concentrations, oxalic acid is present chiefly in the iron(III) complex at pH 2 but in the copper(II) complex at pH 6. When iron(III) at

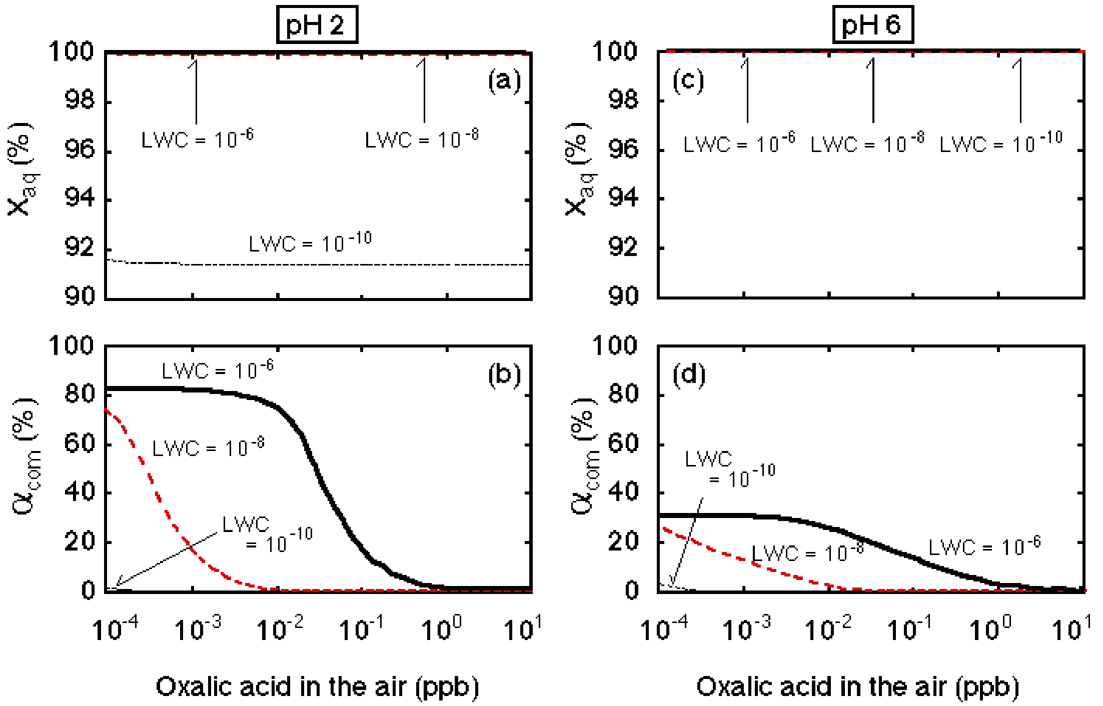

**FIGURE 3**. The dissolution of oxalic acid gas into fog water in the presence of some trace transition: (a) the partition of oxalic acid between gas phase and fog water with pH 2; (b) the fraction of metal–oxalate concentration to total oxalic acid concentration in fog water with pH 2; (c) the partition of oxalic acid between gas phase and fog water with pH 6; (d) the fraction of metal–oxalate concentration to total oxalic acid concentration in fog water with pH 6. The calculation is based on the annual concentration of metals in fog water collected in Mt. Oyama, Japan in 1998, namely $[Fe]_T$ = 764 nM, $[Mn]_T$ = 196 nM, $[Ni]_T$ = 72 nM, $[Cu]_T$ = 175 nM, $[Zn]_T$ = 1349 nM, and $[Pb]_T$ = 102 nM.

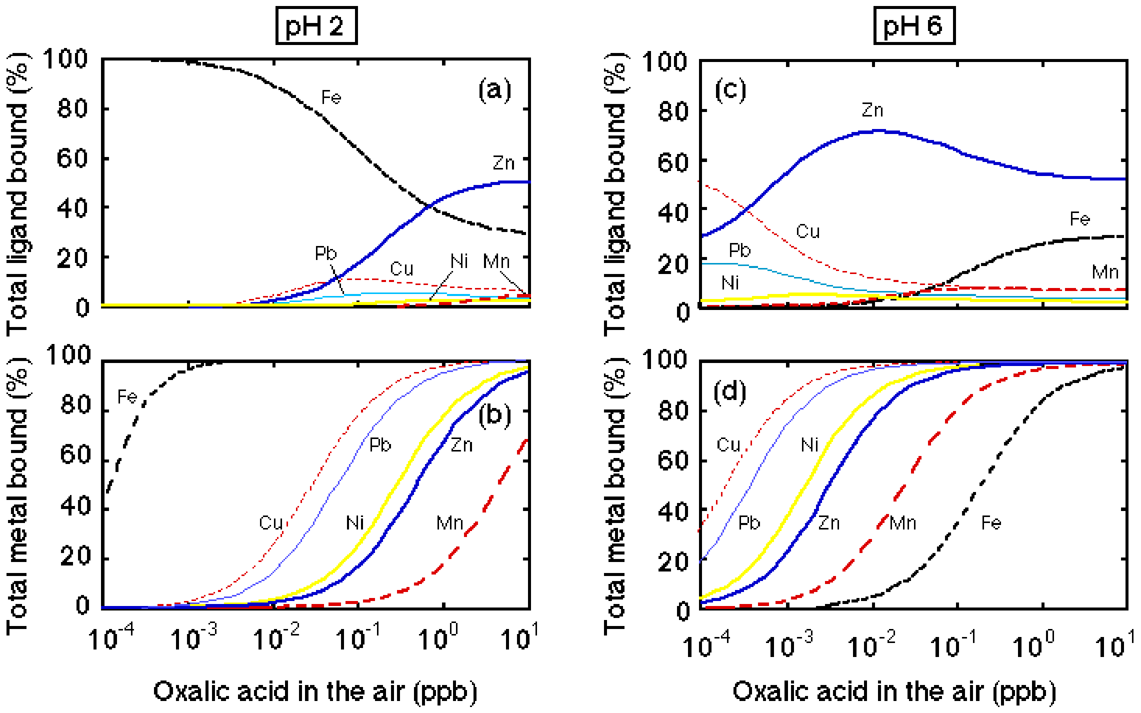

**FIGURE 4**. Speciation of oxalic acid and metals in fog water: (a) speciation of oxalic acid in fog water with pH 2; (b) speciation of the metals in fog water with pH 2; (c) speciation of oxalic acid in fog water with pH 6; (d) speciation of the metals in fog water with pH 6. The calculation is made at LWC $10^{-8}$ and the same concentrations of the metals as those in Fig. 3.

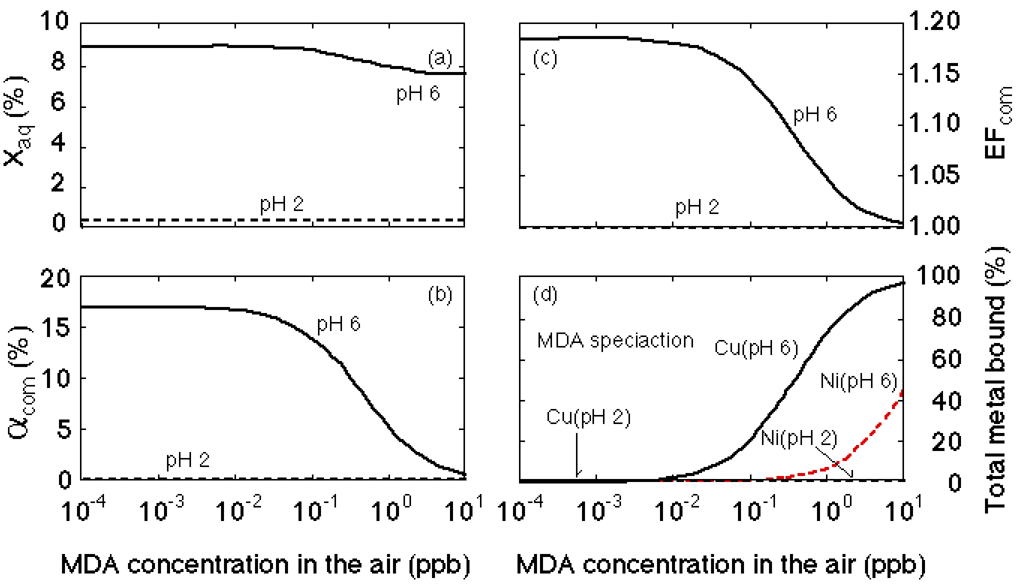

**FIGURE 5**. The dissolution of MDA into fog water in the presence of copper(II) and nickel(II): (a) the partition of MDA between gas phase and fog water with pH 2 and 6; (b) the fraction of metal-MDA concentration to total MDA concentration in fog water with pH 2 and 6; (c) the enhanced uptake of MDA into fog water with pH 2 and 6; (d) speciation of the metals in fog water with pH 2 and 6. The calculation is made at LWC $10^{-8}$, $[Cu]_T = 22.0$ μM and $[Ni]_T = 1.42$ μM.

pH 2 or copper(II) at pH 6 are almost completely complexed as the oxalic acid concentration exceeds those of iron(III) or copper(II) in fog water, the zinc(II) complex becomes significant at both pH conditions (Fig. 4a and 4c). However, the effect of the zinc(II) complex on gas–aqueous phase partition of oxalic acid is small at pH 2 because the zinc(II) concentration is already substantially lower than the oxalate concentration when the zinc(II) complex becomes important (Fig. 3b and Fig. 4a). Other metals contribute little to the partition of oxalic acid, although the contribution of lead(II) might be relatively high in fog water with higher pH and at very low concentrations of oxalic acid (Fig. 4c). Although some other dicarboxylic acids, hydroxy-, and oxocarboxylic acids (notably malonic, tartaric, and citric acids) show substantial complexation in fog water, they also have large Henry's law constants, so enhancement of partition into the aqueous phase is not expected.

By contrast we estimated the gas–aqueous phase partition of MDA, which has only a moderate Henry's law constant. As listed in Table 1, available data for the stability constants of MDA are limited to nickel(II) and copper(II), so we evaluated the effect of both metal ions on the partitioning. We here define an enhancement factor for partitioning via complexation of MDA with metal ions ($EF_{com}$) as the ratio of the aqueous fraction of organic compounds in the presence of metal ions to that in the absence of metal ions:

$$\begin{aligned} EF_{com} &= X_{aq}\,(\text{complexation}) / X_{aq}\,(\text{noncomplexation}) \\ &= (K_H^* / K_H') \times \{(1+ K_H'\, RT \bullet LWC) / (1+ K_H^*\, RT \bullet LWC)\} \end{aligned} \quad (12)$$

where $K_H'$ and $K_H^*$ are the operational Henry's law constant in the absence and presence of metal ions in the aqueous phase, i.e., $K_H' = K_H (1 + K_{a1}/[H])$ and $K_H^* = K_H \{1 + K_{a1}/[H](1 + K_{CuL}[M] + K_{NiL}[M])\}$, respectively. Fig. 5 illustrates the $EF_{com}$ as a function of gas-phase MDA concentration before the formation of fog. The partition of MDA between gas phase and fog water, $X_{aq}$, speciation of MDA in fog water, $\alpha_{com}$, and speciation of metals in fog water are also shown. We here use $[Cu]_T$ = 22.0 μM and $[Ni]_T$ = 1.43 μM, which are the maximum concentrations in fog water listed in Fig. 2, and LWC $10^{-8}$ to estimate the effect of the complexation on the gas–aqueous partition of MDA. As shown in Fig. 5a, MDA is present at about 9% in the aqueous phase at pH 6 and LWC $10^{-8}$. The enhanced uptake of MDA into aqueous phase via the complexation is expected to be 20% in situations where MDA concentration does not exceed the metal concentration (Fig. 5b, 5c, and 5d). The contribution of the copper(II) complex to total metal complexes is almost 100%, although it is not shown in the figure. Using the annual average and the maximum concentration of copper and nickel in fog water at Mt. Oyama, the enhanced uptake of MDA via the complexation is estimated to be 0.2 and 3% in the same condition, respectively. However, there is great potentiality that the uptake of MDA could be more enhanced in the presence of other transition metals such as iron(III) and zinc(II). At pH 2, below its acid-dissociation constant, i.e., pKa 4.7, MDA is almost present in the gas phase, and the effect of the complexation on the partition is negligible (Fig. 5a, 5b, and 5c) because MDA is mostly present in the protonated form in this pH range.

To sum up, the complexation could enhance the gas–aqueous partition of gaseous atmospheric ligands that have moderate Henry's law constants, at moderate low pH, which is not below the first acid-dissociation constant of the ligands, and in situations where the ligand concentrations are substantially lower than the metal concentrations.

## CONCLUSIONS

Macromolecular ligands may form the strongest complexes with trace metals, in particular copper(II), in the atmospheric droplets[3]. The smaller polycarboxylic acids are also likely to be found as metal complexes. In particular, it would appear that dicarboxylic acids such as oxalic, malonic, and phthalic acids would complex with aqueous transition metal ions. Hydroxylated di- and tricarboxylic acids such as tartaric and citric acid would also be complexed with metals. This renders these polycarboxylic acids sensitive to photodegradation. By contrast, the larger dicarboxylic acids, e.g., succinic or pinic acids, are not likely to be so strongly bound and may thus be protected from photochemical degradation in the liquid phase.

Although it is not clear from this research that the complexation can enhance dissolution of metals from solids or mineral phases, it influences chemical speciation and the distribution of both metals and organic compounds in aqueous systems in the atmosphere. Complexation may enhance the dissolution of metals; for example, the solubility of iron(II) compounds, which is known to form through the photoreduction of iron(III)–organic complexes in atmospheric water (e.g., [40]), is higher than that of iron(III) compounds. Complexation could enhance partition of likely atmospheric ligands into the aqueous phase. However, the dicarboxylic acids are already strongly partitioned into atmospheric aerosols. Enhancement of partition may be relatively rare because it requires a special set of circumstances: (1) the metal concentration must be similar to the dissolving ligand at least, (2) the ligand must have a modest Henry's law constant (so that it will not partition regardless of complexation), and (3) the acidity of the ligand must be strong to dissociate at atmospheric pH values. A candidate for this behavior might be malondialdehyde (MDA), although it is only known as yet from smog chamber experiments, and trial calculations for Mt. Oyama fog water suggest a relatively modest enhancement (3%) at best.

Our simple analysis has avoided systems with many ligands or indeed a mixture of metals that is likely in reality. This does complicate the analysis. Given the roles that complexation may play in droplet-phase chemistry, it is worthwhile to improve our knowledge of ligands that might complex with metals in the atmosphere.

## ACKNOWLEDGMENTS

We would like to thank Prof. M. Igawa at Kanagawa University, Japan for allowing us use of analytical data of fog water; Dr. Mario Massucci in the British Antarctic Survey, NERC, U.K. for valuable comments; and Ms. Liz Rix at the University of East Anglia, U.K. for analysis of oxalate by ion chromatography. We thank three reviewers for their helpful comments.

## APPENDIX
## DETERMINATION OF STABILITY CONSTANTS FOR COPPER(II) GLYOXAL COMPLEXES

The $\alpha$-dicarbonyls exist mainly as their hydrated glycols in aqueous solution[A1]. A large hydration constant of glyoxal of $K_{HYD}$ 7.22 × $10^4$[A2], typical of many carbonyls, is responsible for this. The low remaining concentration of unhydrated $\alpha$-dicarbonyl makes it difficult to measure the stability constants with metals.

### Materials

A 40% reagent-grade aqueous solution of glyoxal (Aldrich) was used either without any purification or after passage through a Cl-form ion exchange column to eliminate possible anionic contaminants. The glyoxal was periodically standardized[A3] without filtration. All measurements were made below 0.01 M glyoxal to avoid dimerization[A4]. Oxalic acid, used to evaluate the reliability of our method, was prepared from AnalaR-grade oxalic acid dihydrate (BDH), standardized by alkalimetry. Buffer solutions (0.02 M) were prepared with AnalaR-grade sodium acetate anhydrate (BDH) and glacial acetic acid (FISONS). An acetate buffer system was used because of its low affinity with copper ($\log K_{ML} = 1.8$)[A5]. Stock solution of cupric ion was prepared from 1,000-ppm standard solution of copper for ICP-AES (BDH). Deionized water (18 MΩ cm) was obtained from a Millipore MilliQ system.

### Methods

Copper was titrated into the glyoxal solutions and monitored with cupric ion–selective electrode (Orion ionplus Design 9629BN/Jenway pH meter 3310). Ionic strength was adjusted to 0.10 with 1.0-M $KNO_3$ solution. The jacketed titration vessel was kept at 25˚C by circulating water and was covered with aluminium foil to prevent photolysis. It was continuously stirred with a Teflon®-covered stirring bar. The observed slope was 30.2 ± 1.0 mV per tenfold change in cupric ion concentration. Potentials were read 5 min after each addition of cupric ion. This equilibration was based fixed in a preliminary study. The $Cu^{2+}$ concentration was determined from a calibration.

### Results

Assuming cupric ions form a 1:1 complex with unhydrated glyoxal, it is possible to estimate the stability constant adopting:

$$[Cu^{2+}]/[CuL] = [Cu^{2+}]/[L]_{Total} + 1/(K_{CuL}\,[L]_{Total}) \quad (13)$$

which is frequently used to measure the complexing capacity in natural waters (see [3] and references therein). Here $[Cu^{2+}]$ is the concentration of free cupric ion, [CuL] the concentration of copper bound to the ligand, $[L]_{Total}$ the total ligand concentration, and $K_{CuL}$ is the conditional stability constant. A plot of $[Cu^{2+}]/[CuL]$ as a function of $[Cu^{2+}]$ is linear, provided only a single ligand complexes with the metal. This allows $[L]_{total}$ to be calculated from the reciprocal of the slope and $K_{CuL}$ from the slope/y-axis intercept.

The analysis of measurements on oxalic acid solutions (1.4 and 9.6 μM) gave $\log K_{CuOxa}$ 5.63 and 5.67, respectively, at 25˚C and I = 0.10 M (Fig. 6a). These can be extrapolated to I = 0 as 6.5 and 6.6, close to the values in Table 1, showing that the method is reliable. The stability constants

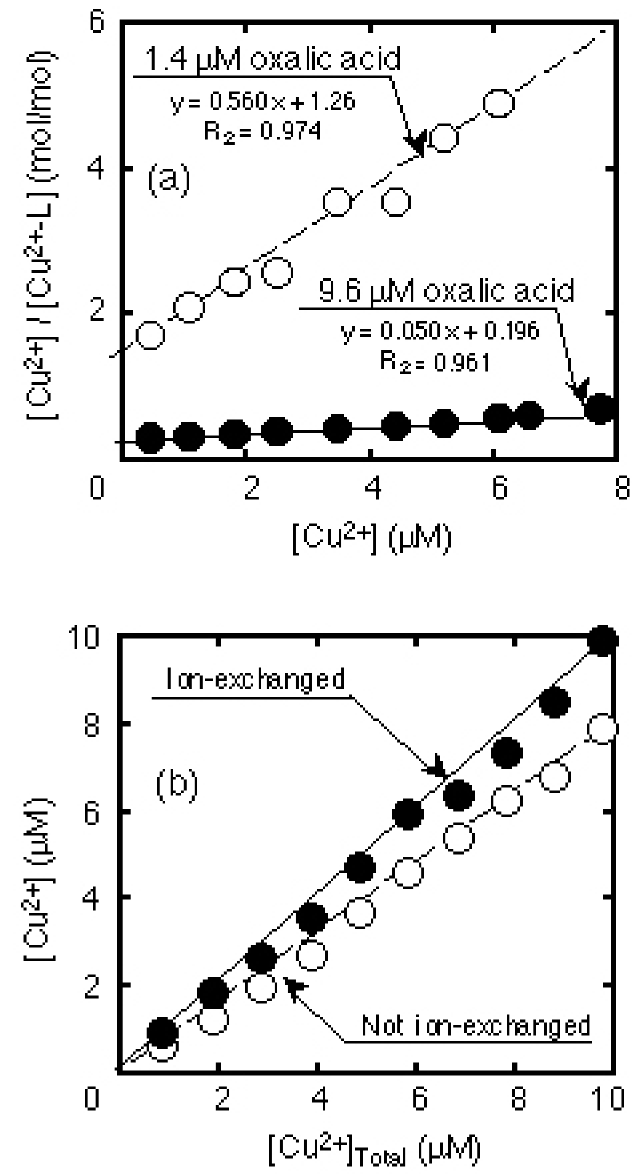

**FIGURE 6**. (a) Linearization of 1.4- and 9.6-μM oxalic acid solution using Eq. 13. Ligand concentrations are estimated from the inverse of the slope and stability constant from the y-axis intercept. (b) The relationship between total and free cupric ion concentration in the presence of 0.1-M glyoxal, which was treated with and without Cl-form ion-exchanged column.

in unpurified 0.01-M glyoxal were determined in triplicate at pH 4.0, 5.0, and 6.0 and gave log $K_{CuGlx}$ 5.25 ± 0.03, 5.38 ± 0.12, and 5.21 ± 0.04 and log $K_{HYD}$ of 4.68 ± 0.18, 4.59 ± 0.13, and 4.62 ± 0.12 (compare with glyoxal of log $K_{HYD}$ 4.86 from Montoya and Mellado)[A2]. Fig. 6b shows $[Cu^{2+}]_{Total}$ equal to $[Cu^{2+}]$, which has a slope of 1 for the purified glyoxal (black dots), indicating that there is no evidence of complex formation. We found the traces of oxalic acid included in unpurified glyoxal solution as contaminants by analyzing it with ion chromatography (1.2 μM oxalic acid was detected in 0.1-M unpurified glyoxal solution). It seems likely that the slope of less than one in Fig. 6b for unpurified glyoxal is due to oxalic acid impurities. Our results provide no evidence for complexation of either unhydrated or indeed the hydrated form of glyoxal with cupric ion.

A1. Wasa, T. and Musha, S. (1970) Polarographic behavior of glyoxal and its related compounds. *Bull. Univ. Osaka Pref. Ser. A* **19**, 169–180.
A2. Montoya, M.R. and Mellado, J.M.R. (1995) Hydration constants of carbonyl and dicarbonyl compounds: comparison between electrochemical and no electrochemical technique. *Port. Electrochim. Acta* **13**, 299–303.
A3. Salomaa, P. (1956) Two volumetric methods for the determination of glyoxal. *Acta Chem. Scand.* **10**, 306–310.
A4. Montoya, M.R. and Mellado, J.M.R. (1992) An electrochemical evidence of the dimerization of methylglyoxal in concentrated aqueous solutions. *Gazz. Chim. Ital.* **122**, 171–173.
A5. Yasuda, M., Yamazaki, K., and Ohtaki, H. (1960) Stability of complexes of several carboxylic acids formed with bivalent metals. *Bull. Chem. Soc. Jpn.* **33**, 1067–1070.

## BIOSKETCHES

**Hiroshi Okochi** is a Research Associate in Kanagawa University. He holds the degree of Doctor of Engineering in Environmental Chemistry. His research interests are in chemistry of atmospheric water (rain, fog, and dew) and the effect of air pollutants on the environment, in particular forested ecosystem.

**Peter Brimblecombe** is Professor at the University of East Anglia, U.K. He holds a Ph.D. in Chemistry. His research interests are in the thermodynamics of aqueous aerosols, pollution damage, damage to cultural materials, and the history of air pollution.
